# Differentiation and Application of Human Pluripotent Stem Cells Derived Cardiovascular Cells for Treatment of Heart Diseases: Promises and Challenges

**DOI:** 10.3389/fcell.2021.658088

**Published:** 2021-05-12

**Authors:** Yu Gao, Jun Pu

**Affiliations:** Department of Cardiology, Ren Ji Hospital, School of Medicine, Shanghai Jiao Tong University, Shanghai, China

**Keywords:** human pluripotent stem cells (hPSCs), cardiovascular cells, differentiation, therapeutic application, large animal

## Abstract

Human pluripotent stem cells (hPSCs) are derived from human embryos (human embryonic stem cells) or reprogrammed from human somatic cells (human induced pluripotent stem cells). They can differentiate into cardiovascular cells, which have great potential as exogenous cell resources for restoring cardiac structure and function in patients with heart disease or heart failure. A variety of protocols have been developed to generate and expand cardiovascular cells derived from hPSCs *in vitro*. Precisely and spatiotemporally activating or inhibiting various pathways in hPSCs is required to obtain cardiovascular lineages with high differentiation efficiency. In this concise review, we summarize the protocols of differentiating hPSCs into cardiovascular cells, highlight their therapeutic application for treatment of cardiac diseases in large animal models, and discuss the challenges and limitations in the use of cardiac cells generated from hPSCs for a better clinical application of hPSC-based cardiac cell therapy.

## Introduction

Cardiovascular diseases are the leading causes of death in the world. It is estimated that more than 5 million people die of myocardial infarction (MI) every year (Virani et al., 2020). Although thrombolysis, coronary intervention, and coronary artery bypass graft have significantly improved the prognosis, the high morbidity and mortality associated with MI indicate that current treatment strategy is far from satisfactory.

Cell transfer therapy is being explored as a potential approach to repopulate damaged cardiac tissue. In addition to skeletal myoblasts (Ye et al., 2007), bone marrow–derived cells (Assmus et al., 2010), and mesenchymal stem cells (Ye et al., 2013a), pluripotent stem cells (PSCs)–derived cardiovascular cells, including cardiovascular progenitor cells (CPCs), cardiomyocytes (CMs), endothelial cells (ECs), and smooth muscle cells (SMCs), have been extensively studied.

Human PSCs (hPSCs) include human embryonic stem cells (hESCs) and human induced PSCs (hiPSCs). Theoretically, they can differentiate into all somatic cells found in the human body and can be used as a disease model to explore the genetic mechanisms of diseases such as congenital heart defects (Lin et al., 2021) or test drugs (Protze et al., 2019) or alternative cell sources for replacing diseased or damaged tissue or disease models *in vitro*. A variety of protocols have been developed to differentiate hPSC into cardiovascular lineages *in vitro*. In this review, we focus on ongoing progress in hPSC-based strategies for human cardiovascular cell derivation and application. This review summarizes the available protocols of differentiating hPSCs into cardiovascular cells, including CMs, ECs, SMCs, and CPCs, and highlights their therapeutic application for treatment of heart diseases in large animal models. Finally, the review discusses the challenges and limitations in the use of cardiac cells generated from hPSCs in the clinical perspective for the treatment of cardiac disease.

## Embryonic Heart Development

**FIGURE 1:**
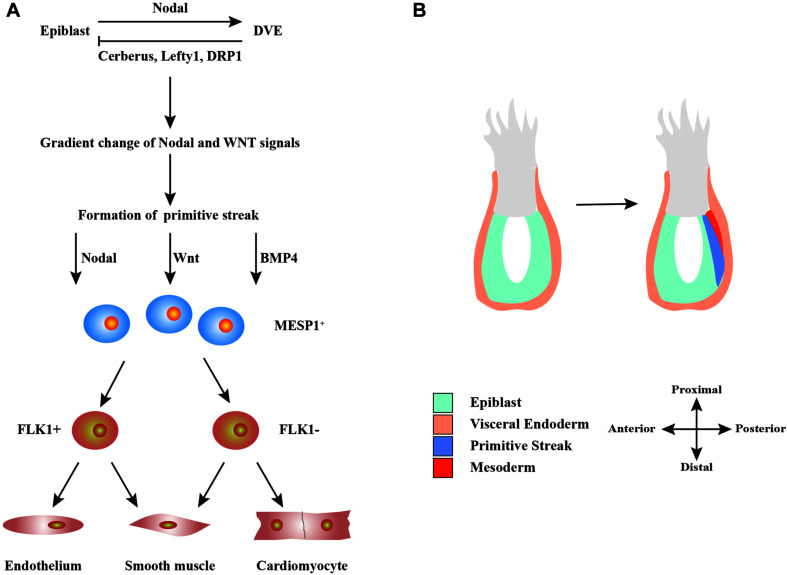
Schematic diagram of the development of heart cells *in vivo*. **(A)** Mutual regulation between epiblast and distal visceral endoderm (DVE) through Nodal, Cerberus, Lefty1, and DRP1 signals leads to a gradient distribution of the concentrations of Nodal and WNT, which results in the formation of primitive streak. During primitive streak migration, a small number of cells express mesoderm posterior protein 1 (MESP1), marking the beginning of heart development. MESP1^+^ cells finally differentiate into various cells that form the heart, such as endothelium, smooth muscle, and myocardium. **(B)** The migration of the primitive streak from posterior to anterior also marks the beginning of gastrulation, a crucial event in embryonic development. During this period, the embryo becomes a trilaminar embryonic disk, and the heart develops from the mesoderm.

The differentiation of hPSCs into CMs is similar to the process of the heart development and formation *in vivo* (Figure 1). Detailed signaling and transcriptional networks in heart development were described in a review by Bruneau (2013). During the embryonic period, Nodal is expressed in the epiblast and activates the distal visceral endoderm, which moves toward the oval to form the anterior–posterior axis (Perea-Gomez et al., 2002). Simultaneously, visceral endoderm secretes Nodal antagonists, including Cerberus, Lefty1, and Dickkopf-related protein 1, which make a gradient change of Nodal and WNT signals in the front and rear directions (Perea-Gomez et al., 2002). This promotes the development of primitive streak, which indicates the start of gastrulation, a process in which the inner cell mass is converted into the trilaminar embryonic disk (Perea-Gomez et al., 2002). This disk comprised the three germ layers: ectoderm, mesoderm, and endoderm. The induction of cardiac mesoderm and distinct populations of CPCs are primarily controlled by three families of extracellular signaling molecules: wingless integrated (WNT), fibroblast growth factor (FGF), and transforming growth factor β (TGF-β), including WNT3a, bone morphogenetic protein 4 (BMP4), Nodal, and activin-A (Spater et al., 2014). These signals induce the expressions of Brachyury and Eomes, which are markers of early mesoderm formation (Lim and Thiery, 2012). In the process of primitive streak migration, cells temporarily activate the transcription factor mesoderm posterior protein 1 (MESP1), which indicates entering the stage of cardiac mesoderm development (Lim and Thiery, 2012). Later, mesodermal progenitors commit to cardiac cells by WNT antagonist.

A subset of MESP1^+^ cells begin to transcribe the homeodomain transcription factors Nkx2.5, T-box 5 (Tbx5, a marker of the first heart field), and islet1 (Isl1) genes (a marker of the second heart field) (Evans et al., 2010). These factors represent cardiac lineage markers in the early developmental stages of the heart field. Nkx2.5 and Tbx5 are typical markers of primitive heart tube cells involved in the formation of the atria and left ventricular (LV) compartments, whereas the secondary heart field is mainly related to the development and formation of the right ventricle and outflow tract (Evans et al., 2010). They are related to transcription factor GATA4/5/6 and serum response factor (SRF). Subsequently, genes related to the CMs are successively activated, such as α-actinin, myosin light chain, myosin heavy chain (MHC), and troponin, as well as myocyte enhancer factor-2 (MEF2) that regulates heart structural genes (Evans et al., 2010). These complexes process and lead to the proliferation and maturation of CMs.

In summary, heart development can be roughly divided into three stages: (1) gastrulation to cardiac specification during which mesodermal progenitors are developed; (2) heart development before beating during which cardiac progenitors are developed; and (3) heart development at beating during which myofibrillogenesis and trabeculation are developed.

## Differentiation of hPSCs Into CMs

### Signaling Pathways Involved in Differentiation of hPSCs Into CMs

The basic principle of the current method of inducing hPSCs to differentiate into CMs *in vitro* is to simulate the heart development *in vivo*. The same differentiation regulation has been demonstrated in hESCs and hiPSCs. hPSCs are differentiated into CMs based on three stages through spatial–temporal modulation of signaling pathways, such as BMP, activin-A, WNT, etc. (Filipczyk et al., 2007; Laflamme et al., 2007; Kattman et al., 2011; Lian et al., 2012; Zhang et al., 2012; Fonoudi et al., 2015).

BMP-4 commits hPSCs into mesodermal lineage cells alone or in combination with activin-A. BMP signaling controls the expressions of GATA4, SRF, and MEF2C transcription factors (Klaus et al., 2012). Combination of activin-A and BMP4 induces KDR^+^PDGFRα^+^ cardiogenic mesoderm in hPSCs, which expressed MESP1 between days 3 and 4 and Nkx2.5 by day 8 of differentiation (Kattman et al., 2011). Combining activin-A and BMP-4 with Matrigel-generated high purity (up to 98%) and yield (up to 11 CMs/input PSC) of CMs from hPSCs (Zhang et al., 2012).

WNT plays a bidirectional role in differentiation, depending on the time point of differentiation. At stage 1, both classical activation, which suppresses the catenin/GSK3 pathway, and non-classical signal transduction, which involves the C protein kinase C/C-Jun N-terminal kinase, have been shown to induce mesodermal lineage from hPSCs (Cohen et al., 2008). At stage 2, WNT antagonist, such as DKK1 and IWP, directs mesodermal progenitors to cardiac progenitors (Willems et al., 2011). Nkx2.5, Isl1, and Baf60c are controlled by WNT/β-catenin signaling (Klaus et al., 2012).

Although either WNT activation/GSK3 inhibition (Ye et al., 2013b; Tan et al., 2019; Tao et al., 2020) or BMP4/activin-A (Laflamme et al., 2007; Hudson et al., 2012; Zhang et al., 2012; Ye et al., 2013b) has been shown to induce mesodermal lineage from hPSCs, WNT activator alone, such as CHIR99021, has gained popularity because of its cheap price and reproducible results (Lian et al., 2012; Su et al., 2018; Tan et al., 2019; Tao et al., 2020).

### Three-Dimensional Environment for CM Differentiation

To mimic *in vivo* cardiac cell development, hPSCs were cultured in embryoid bodies (EBs) or spheroids (Itskovitz-Eldor et al., 2000; Kehat et al., 2001; Xu et al., 2002; He et al., 2003; Zhang et al., 2009; Kattman et al., 2011; Fonoudi et al., 2015; Kempf et al., 2015) or in suspended microcarriers (Ting et al., 2014) to differentiate into CMs. Differentiation of hPSCs in EBs results in three embryonic germ layer formation (Itskovitz-Eldor et al., 2000). Early studies showed that the cells in EBs generated spontaneous contraction and contained mixed cell populations of nodal-, atrial-, and ventricular-like cells, and the efficiency was quite low (Kehat et al., 2001; He et al., 2003; Zhang et al., 2009).

The size of EBs seems to be a critical factor that affects differentiation efficiency. Centrifugation (Ng et al., 2005; Burridge et al., 2007), engineered microwells (Mohr et al., 2006, 2010), and micropatterning (Bauwens et al., 2008) have been employed to produce more homogenously sized EBs, which is helpful for maximizing mesoderm formation and cardiac induction (Bauwens et al., 2008). More recently, spatial–temporal modulation of WNT signaling and activation of sonic hedgehog signaling in hPSCs, cultured in stirred suspension bioreactors, led to the generation of approximately 100% beating EBs containing highly pure (∼90%) CMs in 10 days (Fonoudi et al., 2015). Using bioreactors, 4 × 10^7^ to 5 × 10^7^ CMs can be generated per differentiation batch at >80% purity in 24 days (Kempf et al., 2015).

### Two-Dimensional Environment for CM Differentiation

**FIGURE 2:**
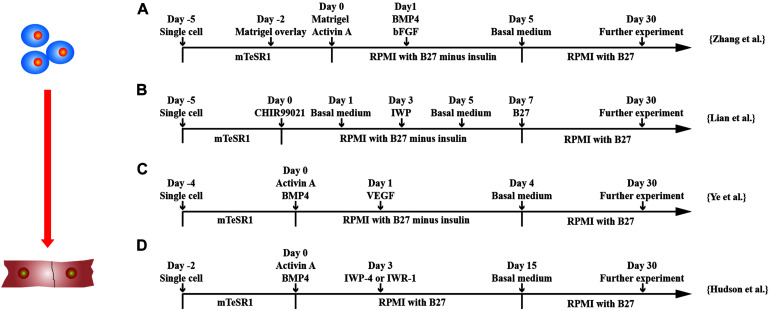
Schematic diagram of differentiation of hPSCs into cardiomyocytes. **(A)** Schematic diagram of matrix sandwich protocol. Extracellular matrix application promoted epithelial–mesenchymal transition of human PSCs. **(B)** Schematic diagram of GiWi small molecule differentiation protocol, which proved that timing regulation of Wnt signal was critical. **(C)** Schematic diagram of activin-A/BMP-4/VEGF protocol, which efficiently differentiated cardiomyocytes from both integrated and non-integrated hiPSCs. **(D)** Schematic diagram of flexible Wnt signal suppression protocol, which indicated that the differentiation of various cell types can be flexibly changed.

Directed differentiation of hPSCs in monolayer is a more convenient method as compared with cardiac differentiation in three-dimensional (3D) environment. Two most efficient and popular CM differentiation methods are the GiWi small molecule differentiation protocol by Lian et al. (2012) and the matrix sandwich method by Zhang et al. (2012) (Figure 2). Lian et al. (2012) showed that temporal modulation of WNT signaling is essential and sufficient for efficient cardiac lineage induction in hPSCs under defined and growth factor–free conditions. WNT activation at the initial stage of hPSC differentiation enhanced CM generation, whereas shRNA knockdown of β-catenin during this stage fully blocked CM specification. Sequential treatment of hPSCs with GSK3, such as CHIR99021, followed by chemical inhibitors of WNT (IWP2) signaling in the later stage produced a high yield (up to 98%) and functional CMs from multiple hPSC lines. Zhang et al. (2012) demonstrated that extracellular matrix also plays an important role in hPSC differentiation. The Matrigel, an extracellular matrix, promotes an epithelial-to-mesenchymal transition, combined with activin-A, BMP4, and basic FGF (bFGF) generated high yield (up to 11 CMs/input PSC) and pure (up to 98%) CMs from hPSCs.

Other factors that affect the differentiation efficiency of hPSCs have been documented, including cell density, cell culture matrices, vascular endothelial growth factor 165 (VEGF_165_), heparin, and insulin. A complete confluence of hiPSCs is required during the differentiation process and increases the yield of CMs (Zhang et al., 2015). In addition to Matrigel, other extracellular matrices, such as recombinant human cadherin, vitronectin, laminin-521 and laminin-511, fibronectin, and a fibronectin mimetic, support efficient CM differentiation of hPSCs (Burridge et al., 2014). A low concentration of VEGF-A at differentiation stage 2 efficiently differentiated hiPSCs into CMs, especially the one reprogrammed from blood mononuclear cells (Ye et al., 2013b). Heparin can act as a WNT modulator to promote CM production condition (Lin et al., 2017). Insulin can redirect differentiation from cardiogenic mesoderm and endoderm to neuroectoderm in differentiating hESCs (Freund et al., 2008).

### Xeno-Free and Chemically Defined Systems for CM Differentiation From hiPSCs

To be safe for clinical application, a xeno-free and chemically defined differentiation system is required. E8 medium and StemMACS iPS-Brew XF medium have been developed as xeno-free media for maintenance and expansion of hPSCs. Vitronectin XF and human recombinant laminin are coating matrix for maintaining hPSC growth and differentiation of hPSCs into cardiac cells (Hayashi and Furue, 2016; Yap et al., 2019). Transferrin has been used to replace B27 in a chemically defined medium for CM differentiation from hiPSCs (Zhang et al., 2020). hiPSC-CMs derived from transferrin-supplemented medium have similar transcriptome and the maturation level compared to those generated in B27 minus insulin medium.

High CM differentiation efficiency using xeno-free and chemically defied system have been reported (Burridge et al., 2014; Tan et al., 2018). Burridge et al. (2014) obtained contractile cell sheets of up to 95% cardiac troponin T (cTNT) CMs using RPMI 1640 medium supplemented with L-ascorbic acid 2-phosphate, recombinant human albumin, and small molecules. Using a bovine serum albumin–free and chemically defined system, Tan et al. (2018) were able to differentiate hPSCs into clinical-grade CMs, which generated greater than 80% cTNT + CMs.

### Purification of hPSC-Derived CMs

Although self-beating immature CMs can be obtained through the above methods, the differentiation efficiency is cell line dependent. There are still many unknown non-CMs, such as undifferentiated hPSCs or cell differentiation into other directions. In order to make the hPSC-CM have therapeutic value, non-CMs need to be removed. A detailed description of strategies for purification of hPSC-CM can be found in review by Ban et al. (2017).

#### Genetic Modification

Genetic method to enrich CM was first developed (Anderson et al., 2007; Xu et al., 2008; Kita-Matsuo et al., 2009; Ma et al., 2011). Cardiac-specific promoter, such as αMHC promoter, with puromycin or neomycin selection gene, was introduced into hESCs to generate stable transgenic cell lines (Xu et al., 2008; Kita-Matsuo et al., 2009). The drug selected CMs were 96% pure and could be cultured for over 4 months. Anderson et al. developed two genetic selection systems: (1) negative selection of proliferating cells with the herpes simplex virus thymidine kinase/ganciclovir gene system and (2) positive selection of CMs expressing a bicistronic reporter: αMHC promoter driven green fluorescent protein (GFP) with puromycin-resistance gene (Anderson et al., 2007). However, only the puromycin method enriched CMs up to 91.5% purity, which was about 2.7-fold that of the negative selection method.

Nkx2.5 is expressed in early cardiac mesoderm cells throughout the left ventricle and atrial chambers during embryogenesis (Evans et al., 2010). GFP was engineered to the Nkx2.5 locus of hESC to facilitate the monitoring CM differentiation (Elliott et al., 2011). Den Hartogh et al. (2016) generated a dual fluorescent reporter MESP1 (mCherry)/Nkx2.5 (GFP) line in hPSC. This enabled the visualization of precardiac MESP1 + mesoderm and their further commitment toward the cardiac lineage through activation of Nkx2.5 (Den Hartogh et al., 2016). Jung et al. (2014) used nodal cell inducer TBX3, coupled with MHC6 promoter–based antibiotic selection, which can obtain 80% of functional sinus pacemaker cells. Although genetic modification seems to improve the purity of hPSC-CMs, it may be more useful for monitoring CM differentiation rather than for purifying hPSC-CMs.

#### Cell Surface Markers

Efforts have been made to identify cell surface markers on CMs. Dubois et al. (2011) screened 370 known CD antibodies and found that signal-regulatory protein α (SIRPα) is a marker specifically expressed on hPSC-CMs. Cell sorting targeting SIRPα can enrich cardiac precursors and hPSC-CMs up to 98% purity. In addition, vascular cell (VC) adhesion molecule 1 has been identified as a cell surface marker for cTnT expressing CMs from 242 antibodies by Uosaki et al. (2011).

Lin et al. (2012) developed a protocol to select CPCs based on cell surface markers during differentiation stages. hPSCs were cultured in EBs and dissociated. The low-KDR/c-Kit^–^ CPCs were isolated by fluorescence activated cell sorting. After culture with VEGF/DKK1, cells were further isolated based on CD166. The CD166^+^ cells were differentiated into CMs, and CD166^–^ cells were differentiated into SMCs.

#### Physical or Chemical Methods

Purification of CMs using a Percoll gradient or metabolic selection has been established. Early study using EBs for hPSC-CM differentiation results in cells from three embryonic germ layers (Itskovitz-Eldor et al., 2000). Percoll density centrifugation can enrich CMs reaching 70% (Xu et al., 2002). Because of the special metabolic mode of CMs, a medium containing lactate without glucose has been used to inhibit the growth of non-CMs, so that only CMs can survive, which increases CM purity up to 99% (Tohyama et al., 2013). Fluorescent molecular beacons targeting the mRNA of MHC6/7 in CMs have been developed to enrich cTnT^+^ CMs up to 97% (Ban et al., 2013).

More recently, it has been shown that synergy between CHIR99021 and concurrent removal of cell–cell contact can massively expand hiPSC-CMs *in vitro* (i.e., 100- to 250-fold) (Buikema et al., 2020). The lymphoid enhancer binding factor/T-cell–specific transcription factor activity and AKT phosphorylation are underlying mechanisms for a synergistic effect. The differentiated hPSC-CMs are often a mixture of several CM subtypes, such as atrial-, ventricular-, and pacemaker-like CMs, which cannot meet the requirements of precision medicine. A comprehensive description of how chamber-specific CMs are produced during development and how atrial-, ventricular-, and pacemaker-like CMs are induced *in vitro*, can be found in review of Zhao et al. (2020).

## Differentiation of hPSCs into ECs

Endothelial cells are a thin layer of specialized cells that directly contact with the blood flow, the circulatory system, and blood throughout the body. Therefore, the function of ECs involves multiple fields of vascular biology, such as nxutrient exchange, immune cell adhesion and migration, and intercellular communication (Lerman and Zeiher, 2005). If ECs are damaged or dysfunctional, it is easy to cause atherosclerosis and other common cardiovascular diseases (Lerman and Zeiher, 2005).

### Embryonic Origins of ECs

The development of blood vessels in the embryo is slightly different from CMs. The initial embryonic blood vessels come from the extraembryonic mesoderm of the yolk sac (Goldie et al., 2008). The progenitor cells differentiate to form a solid cell mass called “blood island,” which will fuse to form a primitive network of tubules known as a vascular plexus. The outer layer of cells gradually becomes flattened to become the most primitive ECs, whereas the inner cells form the primitive hematopoietic stem cells (Goldie et al., 2008). These differentiated blood islands continue to fuse to form the vascular plexus, which is further remodeled to form arteries or veins. In addition, the endothelium of cardiac coronary arteries originates from the sinus venosus through VEGF-C–stimulated angiogenesis during the development of the heart (Chen et al., 2014). The coronary endothelium of interventricular septum is differentiated from the endocardium progenitor cells (Harris and Black, 2010).

### Signaling Pathways in EC Differentiation

hPSCs need to be differentiated into mesodermal progenitor cells by regulating WNT signaling pathway followed by commitment to endothelial lineage principally by VEGF signaling (Su et al., 2018; Wang K. et al., 2020). VEGF is a key growth factor in EC differentiation from hPSCs (Olsson et al., 2006; Nourse et al., 2010). VEGF/VEGF receptor (VEGFR) signaling promotes vascular endothelial differentiation by up-regulating ETV2 expression (Liu et al., 2015). Synergistically using BMP4, FGF2, and VEGF up-regulate the mitogen-activated protein kinase (MAPK) and PI3K pathways to induce early vascular progenitors from hiPSC-derived mesodermal progenitors through regulation of the ETS family transcription factors, ETV2, ERG, and FLI1 (Harding et al., 2017).

ETV2 is a dispensable regulator for vascular EC development. It is expressed in hematopoietic and endothelial progenitors in the yolk sac (Koyano-Nakagawa et al., 2012). ETV2 acts downstream of BMP, Notch, and WNT signaling to regulate blood and vessel progenitor specification. Chromatin immunoprecipitation assay by Liu et al. (2015) showed that ETV2 can bind not only to promoters or enhancers of Flk1 and Cdh5, but also to other genes that perform critical roles in vascular endothelial or hematopoietic cells, including GATA2, Meis1, Dll4, Notch1, Nrp1/2, Flt4, Fli1, RhoJ, and MAPK.

### 3D Environment for EC Differentiation

Similar to CM, 3D and two-dimensional (2D) culture systems have been applied to differentiate hPSCs into ECs (Levenberg et al., 2002; Li et al., 2011; Adams et al., 2013; Sahara et al., 2014; Zhang et al., 2014, 2017; Patsch et al., 2015; Sivarapatna et al., 2015; Gil et al., 2016; Liu et al., 2016; Harding et al., 2017; Su et al., 2018; Wang K. et al., 2020). 3D system includes EB formation or patch-mediated EC differentiation (Levenberg et al., 2002; Li et al., 2011; Adams et al., 2013; Zhang et al., 2014; Sivarapatna et al., 2015; Su et al., 2018).

Early studies cultured hESCs in EBs to facilitate formation of the three embryonic germ layers and purified differentiated ECs by cell sorting based on CD31 (Levenberg et al., 2002; Li et al., 2011; Adams et al., 2013; Gil et al., 2016). ECs can be differentiated from EBs of hESCs under hemangioblast differentiation conditions in two stages (Gil et al., 2016). EBs were cultured with BMP4 for 2 days and dissociated into single cells and cultured with BMP4, VEGF, stem cell factor, thrombopoietin, Flt-3 ligand, and bFGF for another 2 days to obtain ECs. Approximately 37% of hESCs differentiated into ECs as assessed by flow cytometry.

Adams et al. (2013) differentiated hiPSCs in EBs to get ECs. Although only 18% of cells differentiated into ECs, which have biological function to react to proinflammatory factors, such as interleukin 1β (IL-1β), tumor necrosis factor α, and lipopolysaccharide.

To monitor EC differentiation, an hESC cell line was engineered with VE-cadherin promoter-driven GFP EBs (Sahara et al., 2015). BMP4 and a GSK3β inhibitor were applied in an early phase and followed by treatment with VEGF-A and inhibition of the Notch signaling pathway in a later phase for EC differentiation in EBs (Sahara et al., 2015). This resulted in differentiation efficiency up to 50% within 6 days.

Recently, ECs were more efficiently generated from EBs based on the modulation of signaling pathways involved in mesodermal progenitor cells in the early stage and endothelial specification at a later stage (Sivarapatna et al., 2015). Human EBs were first differentiated into mesoderm using BMP-4 followed by dissociation and cultured as monolayer and further treated with VEGF to specify EC fate (Sivarapatna et al., 2015). The differentiation protocol improved EC differentiation efficiency by greater than 50%.

It was found that 3D environment promoted hiPSC differentiation into ECs when hiPSCs were seeded into thrombin–fibrinogen patch (Zhang et al., 2014). 3D environment enhanced EC differentiation through up-regulation of p38MAPK and extracellular signal–regulated kinase 1/2 (ERK1/2) signaling pathways (Su et al., 2018). Synergistically using CHIR99021 with U-46619, a prostaglandin H2 analog that activates ERK1/2 and p38MAPK signaling, not only more efficiently induces mesodermal progenitors in early stage, but also enhances ETV2 transcription factor expression at later stage, which leads to >85% hiPSCs converted to EC fate (Su et al., 2018).

### 2D Environment for EC Differentiation

**FIGURE 3:**
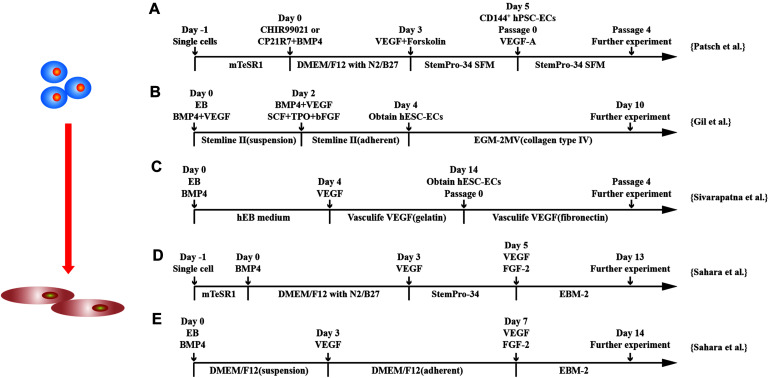
Schematic diagram of differentiation of hPSCs into endothelial cells. **(A)** Schematic diagram of chemically defined protocol. Cells were differentiated to mesoderm by GSK3 inhibition or BMP4 treatment and treated with VEGF to induce ECs. **(B,C)** Schematic diagram of two-stage treated EB protocols. **(D,E)** Schematic diagram of single-cell and EB protocols, respectively, and both methods applied a two-stage cytokine treatment procedure.

With the understanding of signaling pathways required in EC differentiation from hPSCs, 2D monolayer for EC differentiation gains popular. hPSCs are differentiated into intermediate mesodermal progenitor cells at early stage followed by commitment to endothelial specification at later stage by VEGF (Patsch et al., 2015; Sahara et al., 2015; Sivarapatna et al., 2015; Gil et al., 2016; Zhang et al., 2017; Su et al., 2018; Rosa et al., 2019; Wang K. et al., 2020) (Figure 3).

Although a combination of BMP4 and bFGF commits hPSCs into mesodermal lineage (Ikuno et al., 2017; Rosa et al., 2019), synergically using GSK3 inhibitor with BMP4 and/or activin-A has been shown to more efficiently and rapidly commit hPSCs to a mesodermal fate, and subsequent exposure to VEGF-A resulted in efficient differentiation of hPSCs into ECs (Patsch et al., 2015; Sahara et al., 2015; Zhang et al., 2017). CHIR99021 alone has been used for induction of mesoderm at stage 1 of EC differentiation (Liu et al., 2016). At a later stage, VEGF alone or combined with other factors induces EC differentiation from mesodermal progenitors (Patsch et al., 2015). Combining cyclic adenosine monophosphate has been shown to efficiently induced EC differentiation as it increases the expression of VEGFR2 and another VEGFR, neuropilin1, through protein kinase A activation (Yamamizu et al., 2009; Ikuno et al., 2017). Synergistically using FGF2, VEGF, and BMP4 efficiently induced vascular progenitors from hiPSC-derived mesodermal progenitors through regulation of the ETS family transcription factors, ETV2, ERG, and FLI1 via MAPK and PI3k signaling (Harding et al., 2017). Combining VEGF with inhibitor of Notch signaling pathway in the second stage and converted > 50% hPSCs to ECs in 6 days (Sahara et al., 2015).

In addition to small molecules and growth factors, genetic modification has been applied to enhance EC differentiation. Wang K. et al. (2020) transfected mesodermal progenitors with modified mRNA encoding ETV2, a master transcription factor in EC development. This efficiently converted mesodermal progenitors into ECs rapidly and robustly. The implementation of exogenous ETV2 may overcome the issues of inefficient activation of ETV2 during EC differentiation and hiPSCs reprogrammed from various somatic cells.

### Specification of Arterial, Venous, and Lymphatic Endothelial Cells

hPSC-derived ECs are heterogeneous (Rufaihah et al., 2013). They displayed arterial, venous, and, to a lesser degree, lymphatic lineage markers (Rufaihah et al., 2013). The traditional ECs isolated were based on CD31 and/or VE-cadherin, which cannot discern between EC subtypes. Therefore, it is necessary to develop methods to derive or purify iPSC-EC–specific subtypes. Several studies developed protocols to derive more homogenous hPSC-EC subtypes (Rufaihah et al., 2013; Sivarapatna et al., 2015; Zhang et al., 2017; Rosa et al., 2019).

VEGF concentration has been shown to affect differentiated EC subtypes. Rosa et al. (2019) demonstrated that modulation of VEGF concentration (10 vs. 50 ng/mL) can direct mesodermal progenitor cell into venous-like versus arterial-like ECs in a chemically defined and serum-free condition. Rufaihah et al. (2013) confirmed this and further showed that hiPSC-derived ECs are mainly arterial subtype in the presence of high concentrations of VEGF-A (50 ng/mL) and 8-bromoadenosine-3′:5′-cyclic monophosphate (0.5 mmol/L), as they expressed higher levels of ephrin B2, whereas lower concentrations of VEGF-A favored venous subtype and combination of VEGF-C, and angiopoietin-1 promoted the expression of lymphatic phenotype.

Biomimetic flow bioreactors have been employed to facilitate the induction of arterial ECs (Sivarapatna et al., 2015). hiPSC-ECs were purified by CD31^+^ magnetic beads and cultured on bioreactor membrane and ensembled into bioreactors. Flow generated shear stress on hiPSC-ECs, which induced the expressed arterial EC markers: ephrin B2, CXCR4, connexin40, and Notch-1. Zhang et al. (2017) demonstrated that combination of FGF2, VEGFA, SB431542, RESV, and L690 in the absence of insulin greatly improved arterial EC differentiation, whereas venous-like ECs were derived by treating cell with VEGF-A and BMP4 only. The arterial ECs expressed arterial genes, such as CXCR4, DLL4, Notch4, ephrin B2.

There are limited studies on lymphatic endothelial lineage differentiation from hPSCs. Rufaihah et al. (2013) showed that combination of VEGF-C and angiopoietin-1 promoted the expression of lymphatic phenotype. Lee et al. (2015) compared three different culture conditions: spontaneous differentiation through EB formation, coculture with OP9 cells, and a feeder-free culture with gelatin, and found that the coculture system most effectively induced lymphatic endothelial differentiation of hPSCs. Lymphatic ECs expressed key markers, including *PROX1*, *LYVE1*, *VEGFR3*, and *PODOPLANIN*. These cells promoted wound healing through lymphatic neovascularization. More recently, it was shown that low-dose (<1 ng/mL) BMP9 promotes early lymphatic-specified ECs (Subileau et al., 2019).

## Differentiation of hPSCs Into SMCs

The sources of vascular smooth muscle in the embryonic development process are multiple lineages (Majesky, 2007; Sinha et al., 2014), such as neural crest (Jiang et al., 2000), secondary heart field (Waldo et al., 2005), proepicardial organ, lateral plate mesoderm (Mikawa and Gourdie, 1996), and the paraxial mesoderm (Wasteson et al., 2008). Detailed description of the embryonic origins of human vascular SMCs can be found in reviews by Majesky (2007) and Sinha et al. (2014).

Protocol-directed hiPSC-SMC differentiation is quite different in 3D (Xie et al., 2007; Ge et al., 2012; Kinnear et al., 2013; Wang et al., 2014; Kinnear et al., 2020) and 2D (Huang et al., 2006; Patsch et al., 2015; Yang et al., 2016) culture systems. Cells derived from the outgrowth of human EBs cultured in SMC differentiation condition, which was only composed of Dulbecco modified eagle medium (DMEM) + 5% fetal bovine serum (FBS) and a gelatin-coated surface, produced SMCs expressing smooth muscle MHC (SMMHC) and α-smooth muscle actin (α-SMA) (Xie et al., 2007). Surprisingly, when outgrowing cells were cultured in growth condition, which was composed of smooth muscle growth medium and Matrigel-coated surface, <10% of cells expressed SMMHC and α-SMA.

Ge et al. (2012) cultured hiPSC in EBs for 6 days in differentiating medium, which was composed of DMEM medium containing 10% FBS, 1% non-essential amino acids, 0.1 mM mercaptoacids, and 1% L-glutamine. Then, EBs were cultured on 0.1% gelatin–coated surface with fresh differentiation medium for another 6 days. Furthermore, cells were dissociated and transferred to Matrigel–coated plates in SmGM-2 media for 1 week. At last, cells were passaged and cultured on 0.1% gelatin–coated culture dishes and cultured with 5% FBS differentiation medium for at least 5 days to complete differentiation. This produced highly homogenous SMC-like cells (around 96%) (Ge et al., 2012). This protocol has been used to differentiate elastin mutant hiPSC into SMCs to model elastin insufficiency phenotype in SMCs (Kinnear et al., 2020) and Williams–Beuren syndrome *in vitro* (Kinnear et al., 2013). Furthermore, the same protocol has efficiently induced SMCs used for manufacturing of macroporous and nanofibrous poly(L-lactic acid) scaffold (Wang et al., 2014). These studies suggest that human EB mediated SMC differentiation from hPSCs is highly efficient in the condition of DMEM supplemented with FBS and using gelatin as extracellular matrix.

**FIGURE 4:**
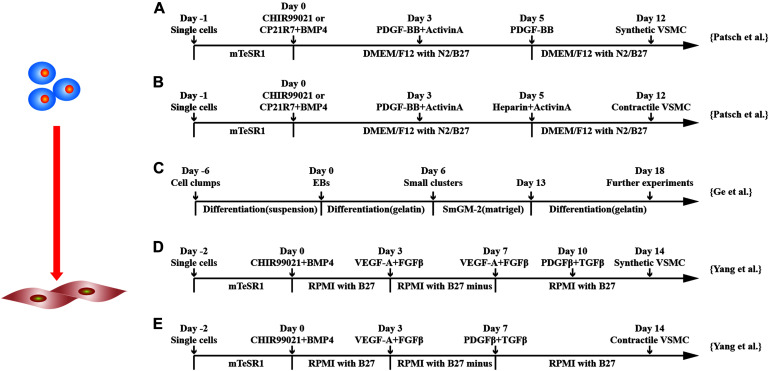
Schematic diagram of differentiation of hPSCs into smooth muscle cells. **(A,B)** Schematic diagram of chemically defined protocols. GSK3 inhibition or BMP4 treatment followed by activin-A and PDGF-BB treatment induced VSMCs from hPSCs, and subsequent applications of PDGF-BB or heparin with activin-A obtained synthetic or contractile VSMCs, respectively. **(C)** Schematic diagram of EB protocol for differentiation of VSMCs from hPSCs. **(D,E)** Schematic diagram of chemically defined protocols that efficiently induced hPSCs to differentiate into VSMCs with different phenotypes. Both methods first used GSK3 inhibition and BMP4 to stimulate differentiation into mesoderm cells and then treated with VEGF-A and FGFβ. Synthetic VSMCs **(D)** were produced by culturing the cells with VEGF-A and FGFβ and with PDGF-β and TGF-β in order. Contractile VSMCs **(E)** were induced by culturing the cells with PDGF-β and TGF-β directly.

In addition to the differentiation through induced EB, monolayer culture differentiation system has been explored (Figure 4). Huang et al. used 10 μM all-*trans* retinoid acid to differentiate hESCs in monolayers. It was shown that > 93% of the cells expressed SMC-marker genes, such as SMMHC and α-SMA, and proteins and were able to contract (Huang et al., 2006). Combining GSK3 inhibition and BMP4 to commit hPSCs to mesodermal cells followed by platelet-derived growth factor two B subunits (PDGF-BB), SMCs can be generated > 80% efficiency within 6 days (Patsch et al., 2015).

Yang et al. (2016) used iPSCs and ESCs from different sources to obtain contractile and synthetic SMC by monolayer cell culture. CHIR99021 and BMP4 were used to induce mesodermal lineage from hPSCs followed by VEGF-A and TGF-β treatment to induce vascular progenitors. Differentiation medium was switched to PDGF-β and TGF-β either on day 7 or 10 to induce contractile or synthetic SMCs. Contractile SMCs expressed higher levels of MHC11 and calponin and had stronger contraction activity, whereas synthetic SMCs expressed more collagen and had stronger proliferation activity. Both protocols converted ∼45% of hPSCs to SMC phenotypes, and the purity could be increased to ∼95% in 4 mM lactate acid in RPMI1640 metabolic medium.

It seems that EB-mediated SMC differentiation can reach similar differentiation efficiency as monolayer differentiation by modulating WNT, PDGF-β, and TGF-β pathways. They may represent SMCs with different lineage background (Majesky, 2007; Sinha et al., 2014). Thus, it is necessary to identify signature markers in SMCs from different lineage and define the lineage specification of SMCs differentiated from protocols. This helps to apply hPSC-SMCs in understanding vascular development in embryo and use disease-specific hPSC-SMCs in disease modeling and drug screening.

## Differentiation of hPSCs Into CPCs

**FIGURE 5:**
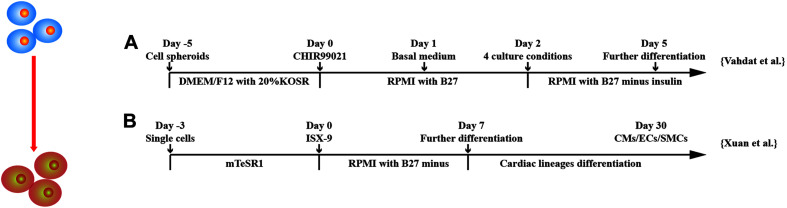
Schematic diagram of differentiation of hPSCs into cardiac progenitor cells. **(A)** MESP1^+^ cells were obtained after treatment of GSK3 inhibition and then were cultured in four different conditions: (a) suspension culture of spheroids, (b) adherent culture of spheroids on gelatin, (c) adherent culture of single cells on gelatin, and (d) adherent culture of single cells on Matrigel. **(B)** Isoxazole (ISX-9), a cardiogenic small molecule, induced CPCs from hPSCs, which further differentiated into three cardiac lineages *in vitro*.

Recently, induction of CPCs is gaining attention as they are able to self-renew and predetermined to differentiate into cardiac lineage cells *in vitro* and *in vivo.* This saves time and is cost-effective as compared to derive CMs, ECs, and SMCs *in vitro* and further transplantation *in vivo*. Various CPC markers, such as stage-specific embryonic antigen 1 (SSEA-1) (Bellamy et al., 2015), MESP1 (Bondue et al., 2008), and Nkx2.5 (Birket et al., 2015), have been investigated (Figure 5).

hPSCs treated with BMP2 give rise to an early population of cardiovascular progenitors, characterized by SSEA-1 (Brade et al., 2013; Bellamy et al., 2015). This progenitor population was multipotential and able to generate CMs, SMCs, and ECs *in vitro*. When purified SSEA-1^+^ progenitors implanted into non-human primates (NHPs), they differentiated into ventricular CMs. However, non-purified SSEA-1^+^ progenitor cell implantation resulted in teratomas in the scar tissue.

Cardiogenic mesodermal cells (CMCs) expressing MESP1 have been shown to differentiate into almost all cardiac cell types both *in vitro* and *in vivo* (Bondue et al., 2008; Brade et al., 2013; Den Hartogh et al., 2015; Lescroart et al., 2018). To monitor early cardiac mesoderm in hPSCs, a dual MESP1 (mCherry/w)–NKX2-5 (eGFP/w) reporter line was developed in hESCs (Den Hartogh et al., 2015). Induction of cardiac differentiation in this reporter line resulted in transient expression of MESP1-mCherry, followed by expression of NKX2.5-eGFP. MESP1-mCherry cells showed increased expression of mesodermal markers. Whole-genome microarray profiling and fluorescence-activated cell sorting analysis of MESP1-mCherry cells showed enrichment for mesodermal progenitor cell surface markers, such as PDGFRα, CD13, and ROR-2. MESP1-mCherry derivatives contained an enriched percentage of Nkx2.5-eGFP and CMs, SMCs, and ECs.

Vahdat et al. (2019a,b) established a protocol for maintenance and large-scale expansion of early CPCs, so-called CMCs in a defined culture system. Through chemical screening, they developed a medium containing three factors, A83-01, bFGF, and CHIR99021, which generated CMCs expressing cardiac mesoderm markers and cardiac-specific transcription factors *MESP1, SSEA1, ISL1, PDGFR*α, *NKX2.5*, and *MEF2c*; 10^14^ CMCs were generated after 10 passages and were able to differentiate into CMs, ECs, and SMCs *in vitro*. To monitor CPC derivation, selection, and maintenance, Birket et al. (2015) engineered hPSCs to carry a cardiac lineage reporter to enable robust expansion of MYC expression primitive pre–NKX2.5^+^CPCs. Through regulation of FGF and BMP signaling, NKX2.5^+^CPCs can be differentiated into ventricular-like cells, pacemaker-like cells, ECs, and SMCs. Yap et al. (2019) developed a chemically defined, xeno-free, laminin-based differentiation protocol to generate CPCs from hESCs. Laminin-221, an abundant laminin isoform in heart extracellular matrix, induced a transcriptomic signature with up-regulated markers for cardiac development. CPCs appeared on day 9 or 11 of differentiation and highly expressed *ISL1*, *TBX5*, *MEF2C*, *C-KIT*, and *GATA3*. Single-cell RNA sequencing of CPCs identified three main progenitor subpopulations, including CMs, SMCs, and small population of epithelial cells. The CPCs generated human heart muscle bundles in mouse heart post–ischemia/reperfusion (I/R) injury. Uosaki et al. (2012) showed that coaggregation of endodermal cell line End2 with hESCs significantly promoted the induction of KDR^+^PDGFRα^+^CPCs, suggesting a direct contact with endoderm-like cells can induce cardiac progenitors from hPSCs. Bylund et al. (2017) found that BMP antagonist GREMLIN 2 is linked to cardiogenesis. Inhibition of canonical BMP signaling followed by JNK pathway activation by GREM2 induced cardiac differentiation of hiPSCs. Furthermore, GREM2 promoted proliferation of CPCs.

Other factors have been shown to be effective in deriving CPCs. Xuan et al. (2018) demonstrated that hiPSCs treated with isoxazole 9 (ISX-9), a potent inducer of adult neural stem cell differentiation, for 3 days stimulated hiPSCs to become CPCs expressing NKX2.5, GATA4, ISL1, and MEF2C and were able to generate CMs, SMCs, and ECs *in vitro* and *in vivo*. ISX-9 activated multiple pathways including TGF-β–induced epithelial–mesenchymal transition signaling and canonical and non-canonical WNT signaling at different stages of cardiac differentiation. Cyclosporin-A, an immunosuppression drug, has been shown to stimulate differentiation of FLK1^+^ mesodermal cells into FLK1^+^/CXCR4^+^/VE-cadherin^–^ CPCs and CMs (Fujiwara et al., 2011). The beating colonies from hiPSCs were increased approximately 4.3 times by addition of cyclosporin-A at mesoderm stage.

One feature associated with hPSC-derived CPCs is their great extracellular vesicle (EV) secretory profile (El Harane et al., 2018). EVs are rich in miRNAs, and most of the 16 highly abundant, evolutionarily conserved miRNAs are associated with tissue-repair pathways. *In vitro*, EV increased cell survival, cell proliferation, and EC migration and stimulated tube formation. *In vivo*, EV significantly improved cardiac function through decreased LV volume and increased LV ejection fraction.

Although CPCs are emerging as a better option as compared to CM transplantation, several issues need to be solved before it can be fully translated into clinic: (1) Purified versus non-purified. Non-purified CPCs have the risk to form teratoma after implantation. It is possible that early CPC population may contain pacemaker cells to behave as foci of automaticity and cause arrhythmias. Thus, purified cell population is preferred. (2) Purification method. Except for SSEA1, MESP1, and NKX2.5 are intracellular markers; if we use genetically modified CPC based on MESP1 or Nkx2.5 expression, there is a concern of safety issue. (3) Electrical coupling: CPCs are not CMs. Although they will develop into CM in heart eventually after implantation, the early developed CMs may cause electrical uncoupling leading to ventricular arrhythmia.

## Cardiomyoplasty in Large Animal Models Using hPSC-Derived Cardiovascular Cells

The continuous improvement of differentiation efficiency of hPSCs has made large quantities of human CPC, CM, EC, and SMC reality. They are being tested as cell transfer therapy for cardiac repair not only in small, but also in large animal heart models of heart diseases (Kawamura et al., 2012, 2013; Xiong et al., 2012; Chong et al., 2014; Ye et al., 2014; Shiba et al., 2016; Gao et al., 2018; Ishigami et al., 2018; Zhu W. et al., 2018; Ishida et al., 2019; Romagnuolo et al., 2019) (Table 1).

**TABLE 1:** Cellular cardiomyoplasty in large animal models using hPSC-derived cardiovascular cells.

Animal model	Cell type	Cell number	Teratoma	Arrhythmia	
Pig model of acute ischemia/reperfusion	hiPSC-CMs hiPSC-ECs hiPSC-SMCs	2.0 × 10^6^ 2.0 × 10^6^ 2.0 × 10^6^	N.A.	Not detected	Ye et al., 2014
Pig model of acute ischemia/reperfusion	hiPSC-CMs hiPSC-ECs hiPSC-SMCs	4.0 × 10^6^ 2.0 × 10^6^ 2.0 × 10^6^	N.A.	Not detected	Gao et al., 2018
Pig model of acute ischemia/reperfusion	hESC-ECs hESC-SMCs	2.0 × 10^6^ 2.0 × 10^6^	N.A.	During surgery	Xiong et al., 2012
Pig model of chronic ischemia	hiPSC-CMs	2.5 × 10^7^	Not detected	N.A.	Kawamura et al., 2012
Pig model of chronic myocardial infarction	hiPSC-CMs hiPSC-ECs hiPSC-VMCs	1.0 × 10^7^	Not detected	Not detected	Ishigami et al., 2018
Pig model of chronic myocardial infarction	hiPSC-CMs	1.0 × 10^8^	N.A.	N.A.	Ishida et al., 2019
Monkey model of chronic ischemia	hESC-CMs	1.0 × 10^9^	Not detected	Within 24 h after delivery	Chong et al., 2014
Monkey model of chronic ischemia	hiPSC-CMs	4.0 × 10^8^	N.A.	Within 4 weeks after delivery	Shiba et al., 2016

*N.A. means that there was no relevant data or information in the article.*

Two large animal models, pig and NHP, have been used as preclinical models to investigate feasibility, efficacy, and safety of hiPSC-derived cardiac cells. Among them, more studies used pigs as the pig’s heart is very similar to human’s in terms of morphology, size, electrophysiology, and metabolic physiology (Lelovas et al., 2014). Comparatively, relatively fewer studies with NHPs have been reported. Although NHPs are more closely resembled to human anatomy, physiology, function, and metabolism (Cox et al., 2017) and are more valuable from an experimental perspective, they are not cost-effective and are associated with ethical issues (Zhu K. et al., 2018; Cong et al., 2019).

### Transplantation of hPSC-CMs Only

Cardiac cells differentiated from hiPSCs have been either directly intramyocardially injected or applied epicardially using cells sheets or patches. In many studies, transplantation of hPSC-CMs into cardiovascular disease model animals could improve heart function and reduce the ventricular remodeling. Kawamura et al. (2012) generated hiPSC-CM sheets using 6-cm thermoresponsive dishes. The cell sheets were approximately 30- to 50 μm thick. Eight hiPSC-CM sheets were implanted through median sternotomy with chronic MI. The transplanted hiPSC-CM sheets attenuated LV remodeling and increased neovascularization without teratoma formation. To enhance survival of hiPSC-CMs posttransplantation, the same group implanted hiPSC-CM sheets with an omentum to enhance blood supply to cell sheets (Kawamura et al., 2013). Histology showed the transplanted tissues contained abundant cTnT^+^ cells surrounded by vascular-rich structures. In addition, it has been found that the transplantation of hESC-CMs can promote remuscularization to a certain extent in both pig and NHP models (Chong et al., 2014; Romagnuolo et al., 2019).

### Transplantation of Multilineage Cardiac Cells

In addition of CMs, VCs, including ECs and SMCs, differentiated from hESCs (hESC-VCs) or hiPSCs (hiPSC-VCs), have been investigated. Implantation of VCs promoted survival of ischemic cells, angiogenesis, and antiapoptotic effect through paracrine factors released. Implantation of hESC-VCs or hiPSC-VCs seeded in fibrin/thrombin patch alleviated LV contractile dysfunction and wall stress and improved myocardial energetics (Xiong et al., 2012) and attenuated the reduction of ATP utilization at infarct border zone (Xiong et al., 2013) in porcine heart model of I/R.

On this basis, researchers began to combine these different cells and transplant them into animal models together. Ye et al. (2014) implanted trilineage cardiac cells, including CMs, ECs, and SMCs, derived from hiPSCs in combination with a fibrin patch loaded with insulin growth factor into porcine heart post–acute I/R. The transplantation of trilineage cardiac cells makes the efficacy of cell therapy more comprehensive and effective: hiPSC-CMs regenerated CM, whereas hiPSC-VC improved donor and host CM viability and stimulated neovascularization. Molecular factors having antiapoptotic (angiogenin, angiopoietin, IL-6, matrix metalloproteinase-1, PDGF-BB, TIMP Metallopeptidase Inhibitor 1, urokinase receptor, and VEGF), promoting cell homing (IL-8, monocyte chemoattractant protein-1, Monocyte chemoattractant protein-3, matrix metalloproteinase-9), and inducing cell division (angiogenin, angiopoietin, PDGF-BB, VEGF) properties were identified in paracrine factors released by hiPSC-CMs and hiPSC-VCs.

Gao et al. (2018) manufactured human cardiac muscle patch (hCMP) using 4 million hiPSC-CMs, 2 million each of hiPSC-ECs and hiPSC-SMCs. They implanted two hCMPs into pig heart model of acute MI. The hCMP transplantation was associated with significant improvements in LV function; reduced cardiac apoptosis, infarct size, and myocardial wall stress; and reversed some MI-associated changes in sarcomeric regulatory protein phosphorylation. Ishigami et al. (2018) generated cardiac tissue sheets using simultaneously induced hiPSC-CMs and hiPSC-VCs in temperature-responsive culture dishes. They transplanted four cardiac tissue sheets on the epicardium of infarcted myocardium in a porcine model of chronic MI. Transplantation resulted in significant increases of circumference strain and capillary density and reduction of fibrotic tissue in infarct and border regions after transplantation.

### Limitations and Improvements in Transplantation Treatment of Animal Models

Histological analysis revealed that only a few implanted hiPSC-CMs survived at week 8 after implantation. Thus, the improved cardiac function was achieved mainly through the paracrine factors instead of regeneration of CMs (Kawamura et al., 2012). Therefore, the recovery of heart function involves many aspects, and it is necessary to use multilineage cell transplantation to improve blood vessel supply, inflammation regulation, and metabolism.

To improve cell engraftment and reduce immunogenicity of allogeneic iPSC-CMs, Kawamura et al. (2016) injected allogeneic monkey iPSC-CMs into MHC-matched or non-matched NHPs. The transplantation of allogeneic iPSC-CMs in MHC-matched NHP had increased cell engraftment with less immune-cell infiltration. Shiba et al. (2016) injected 4 × 10^8^ major histocompatibility complex (MHC) matched allogeneic iPSC-CMs into NHPs post–chronic MI. Transplantation of the iPSC-CMs improved heart contractile function at 4 and 12 weeks posttransplantation. Although electrical coupling was established between donor and host CMs as assessed by use of the fluorescent calcium indicator G-CaMP7.09, the incidence of ventricular tachycardia was transiently, but significantly, increased when compared to control animal group. Furthermore, no macroscopic or microscopic tumor formation was detected. Wang et al. (2019) determined the efficacy of hESC-derived CPCs in NHPs. They found that implantation of hESC-CPCs into acutely infarcted myocardium significantly ameliorated the functional worsening and scar formation, concomitantly with reduced inflammatory reactions and CM apoptosis, as well as increased vascularization. Moreover, hESC-CPCs modulated cardiac macrophages toward a reparative phenotype in the infarcted hearts.

## Challenges of hPSC Technology in the Treatment of Cardiovascular Diseases

### Cell Quality

Although various protocols have been developed to induce hPSCs into CPCs, CMs, ECs, and SMCs, which have been extensively evaluated in small and large animal models of heart diseases, there is a lack of commonly accepted standards to evaluate and control the quality of hPSC-derived cardiac cells. This especially applies to hiPSC-derived cardiovascular cells, as the reprogramming may change the genetic stability, and epigenetic memory may compromise therapeutic outcome.

### Immunogenicity of hPSCs and Their Derivatives

The second issue is related to the immunogenicity of hPSCs. It has been shown that hESCs have low expression of MHCI and complete absence of MHCII antigens and costimulatory molecules, such as CD80 and CD86 (Li et al., 2004; Wu et al., 2008). The expression levels of the above molecules in hiPSCs are almost same as those in hESCs (Suarez-Alvarez et al., 2010). Thus, hPSCs may possess immune privilege property. However, increased MHC expression and immunogenicity have been documented after differentiation (Swijnenburg et al., 2005; Suarez-Alvarez et al., 2010). Although immunosuppressive drug regimens can be used to suppress recipients’ immune response to transplanted allogenic hPSC-derived cells, optimal dose and combination of different drugs to achieve minimal drug toxicity are still far from optimization. Universal hESC or hiPSC cell lines, which have human leukocyte antigen (HLA) class I (HLA-I) and II (HLA-II) knock-out (Han et al., 2019; Xu et al., 2019; Wang X. et al., 2020), may be a solution. HLA-I and HLA-II knockout hiPSCs can generate immunocompatible and ready-to-use cardiovascular cells.

### Defects of hiPSCs Derivatives

The third issue is specifically related to hiPSCs. Although hiPSCs can differentiate into “personalized” patient-specific cells and tissues to circumvent both immunogenicity barriers, they may have limited therapeutic potential if they are reprogrammed from patients with diseases caused by genetic mutations. Again, derivatives of universal hESCs or hiPSCs will be a good option for allogeneic transplantation.

### Optimal Cell Types and Numbers for Cardiac Repair or Regeneration

The fourth issue is associated with cell type and cell dosing. Currently, most studies determined the efficacy of one cell type, either hPSC-CMs or CPCs, whereas only a few have compared different stage-specific cardiac cells. Thus, it is hard to provide unequivocal evidence for the superiority of one type over the other. In large animal heart models, transplanted hPSC-CM numbers ranged between 4 × 10^8^ and 1 × 10^9^ (Chong et al., 2014; Shiba et al., 2016; Liu et al., 2018; Romagnuolo et al., 2019). Although from a clinical perspective, a higher number of hPSC-CMs may be more beneficial to cardiac function, a mixed cardiac cell population may be a cost-effective way as compared with pure CM transplantation. Genetic modification hPSC derivatives with genes to enhance their reparability may be a cost-effective option (Tao et al., 2020).

## Author Contributions

YG and JP conceived the design of the work. YG wrote the manuscript with support from JP. Both authors contributed to the article and approved the submitted version.

## Conflict of Interest

The authors declare that the research was conducted in the absence of any commercial or financial relationships that could be construed as a potential conflict of interest.
